# Associations between self-rated health, life satisfaction and physical activity among adolescents in Saudi Arabia

**DOI:** 10.25122/jml-2021-0046

**Published:** 2021

**Authors:** Ali Saad Alsubaie

**Affiliations:** 1. Department of Public Health, College of Public Health, Imam Abdulrahman Bin Faisal University, Dammam, Kingdom of Saudi Arabia

**Keywords:** adolescents; health status, life satisfaction, physical activity, public health

## Abstract

Empirical evidence has shown that health status and life satisfaction are strongly correlated and connected to physical activity. However, no previous research has examined the association of physical activity (PA) with self-rated health (SRH) and life satisfaction (LS) among adolescents in Saudi Arabia. Therefore, a cross-sectional study was conducted among a sample of male adolescents (n=453; age 15–20 years) in Riyadh, Saudi Arabia. The objective was to investigate the association between LS and SRH with PA in adolescents, taking into account socio-demographic variables. A pre-tested validated questionnaire was used to collect data using a stratified sampling technique. An estimated 354 (78.5%) adolescents reported being healthy, and 98 (21.7%) were satisfied with their lives. The logistic regression analysis revealed that adolescents’ SRH was independently associated with their sense of LS (AOR=2.5, 95%CI: 1.5-4.3, P≤0.001). Also, the odds of reporting being healthy increased 2.5 times for being moderate active (95%CI: 1.5-4.3, P<0.001) and 3.4 times for being highly active (95%CI: 2.0-5.8, P≤0.001), as compared to non-active adolescents. Moreover, adjusted logistic regression showed that adolescents’ LS was independently associated with high level of their father’s education (AOR=1.8, 95%CI: 1.2-3.0, P=0.023); studying in private schools (AOR=1.9, 95%CI: 1.3-2.9, P≤0.011) and being highly active (AOR=3.0, 95%CI: 1.6-4.2, P=0.007). SRH and LS are determinants of each other, and both variables were independently associated with PA levels. Programs that increase PA may promote life satisfaction and health status in the adolescent population. Therefore, it is important to promote physical activity amongst them, create a sport-friendly environment, and engage adolescents in physical activity during their free time by broadening access to more sports classes and other forms of assistance. This is also an important method of enhancing adolescents’ life satisfaction and better health.

## Introduction

According to the World Health Organization (WHO), health is defined as a “state of complete physical, mental and social well-being, and not merely being the absence of disease or infirmity” [1], so health is multidimensional, and subjectivity is an aspect of its measurement. Thus, understanding the underlying factors contributing to an individual’s health and well-being is essential for health prevention and public health. Health can be evaluated objectively through clinical parameters and clinical examination and subjectively to reveal self-perceived health. However, self-rated health (SRH) was suggested as a health indicator among adolescents [2]. Self-rated health (SRH) and life satisfaction (LS) reflects an individual’s reflective judgment of the degree to which his or her life is going well. Perceptions that one can have about their health and life may add value to further decisions regarding lifestyle behavior, such as physical activity [3]. Physical activity is an important determinant of people’s health and quality of life and is recognized as an important factor in the primary and secondary prevention of non-communicable diseases. The health benefits of physical activity are well recognized and can no longer be doubted. Several diseases were linked to the lack of physical activity, such as obesity, diabetes, hypertension, coronary heart disease, stroke, and depression [4, 5]. Also, regular participation in physical activity is associated with a range of positive mental health-related outcomes [6]. Physical activity is positively associated with life satisfaction and better quality of life [7–9]. Besides health benefits, physical activity is associated with other benefits in young people. Several studies have shown a positive relationship between increased physical activity levels and academic achievement and cognitive function [10–13]. Furthermore, physical activity is associated with adolescents’ health, well-being, and life satisfaction [14–17]. Despite the importance of these findings, less is known about the relationship between physical activity and life satisfaction and perceived health in Saudi Arabia.

There is a growing global interest in investigating adolescent health and their perspective about life satisfaction and health behaviors and outcomes. The aim of this investigation was to estimate the independent associations of physical activity with life satisfaction and perceived health status among adolescents in Saudi Arabia.

## Material and Methods

### Study design

This cross-sectional study was performed on a sample of male adolescents studying in high schools, with an age range of 15–20 years, residents in Riyadh, the capital city of Saudi Arabia. A multistage stratified random sampling was used, taking into account the class level (the tenth, eleventh and twelfth year of high schools), regions (4 autonomous communities), and type of schools (2 public schools and 2 private schools). Three classes were selected in each school. A self-administered questionnaire was used for data collection. Full details of the study development and methods employed have been described elsewhere [18, 19].

### Sample size and participants criteria

Using a 95% confidence interval (two-sided), α=0.05 margin of error and since no previous study was found, 0.5 prevalence was used.



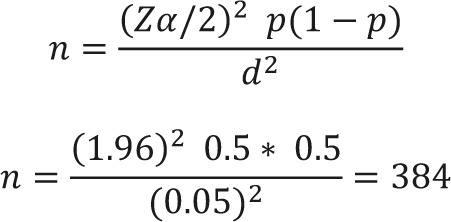



20% is also added to compensate for the non-response rate and other contingencies (missing data).



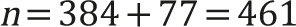



The final sample size included in this study was 453 male adolescent students. Inclusion criteria included high school male adolescent students attending school days in the selected high schools in Riyadh, SA. The following exclusion criteria were used in the study: (1) adolescents who were ill during the time of data collection, (2) disabled adolescents, and (3) adolescents’ who are unwilling to participate.

### Instrument and measurements

The core questions of this study were based on the Health Behavior in School-Aged Children (HBSC) survey by the World Health Organization [20]. The questionnaire was validated through a pilot study. The questionnaire was reviewed in terms of appropriateness and relevance to school students by three experts, professors, and the study researcher. The questionnaire was tested for validity among adolescents (n=23). The researcher explained the aims and objectives of the study to the adolescents , emphasizing that their participation was optional, the importance of honest responses, and assured strict confidentiality of their personal information. Participation in the study was voluntary, and students had a right to refuse to participate in the survey.

Perceived health status was measured with one item, “In general, would you say that your health is: 1 – excellent, 2 – very good, 3 – good, 4 – fair, or 5 – poor”. Later, for regression analysis, adolescents’ health status was dichotomized into “healthy” (a response of excellent/very good/good) and “unhealthy”: (a response of fair/poor). Life satisfaction was evaluated by asking the respondent, “In general, how satisfied are you with your life?”. Responses ranged from 1 – very dissatisfied to 5 – very satisfied. For the binary logistic analysis, adolescents’ life satisfaction was dichotomized to “satisfied” and “unsatisfied”. Physical activity was assessed using the question: “in the last 30 days, on how many days were you physically active for a total of 60 minutes per day?” The response categories were from 0 to 7 days. Outcome variables were broken into three categories: physically inactive (0–2 days/week), moderately active (3–4 days/week), and highly active (≥5 days/week).

### Statistical analysis

Questionnaires were examined for completeness, and adequately completed questionnaires were entered into the computer and analyzed using the SPSS Version 25.0 (Statistical Package for the Social Sciences); (Armonk, NY: IBM Corp.). Only completed questionnaires were included in the analyses. Descriptive statistics, such as frequencies and percentages, were calculated for each item. Adjusted logistic regression analyses, taking into account socio-demographic factors, were performed to investigate the associations between adolescents’ self-rated health and life satisfaction with their physical activity pattern. Adjusted odds ratios (OR) and their 95% confidence intervals (CI) were used as indicators for associative strength. Results were considered significant at a threshold of p <0.05.

## Results

Table 1 presents the participants’ demographic characteristics. The mean age of participants was 17.1 years, a standard deviation (SD) of ±1.33, and a median of 17 years. The age of participants ranged between 15 and 20 years old. A total of 235 (52.1%) of the students’ fathers completed more than high school education, while 130 (28.7%) of their mothers had completed more than high school education.

**Table 1.** Distribution of adolescents’ demographic characteristics.

**Variable**	**No.**	**%**
**Age**		
15–16 years	185	40.8
17–18 years	203	44.8
19–20 years	65	14.3
Total	453	100
**Type of schools**		
Public schools	270	60
Private Schools	183	40
Total	453	100
**Father's educational level**		
≤12 years of education	216	47.9
>12 years of education	235	52.1
Total	451	100
**Mother's educational level**		
≤12 years of education	323	71.3
>12 years of education	130	28.7
Total	453	100
**Family situation**		
Living with both parents	410	91.5
Living with one of them	34	7.6
Living with none of them	4	0.9
Total	448	100

Table 2 shows that 354 (78.5%) of the adolescents rated their health as healthy (excellent health and good health), and 98 (21.5%) rated themselves as unhealthy. Also, a total of 98 (21.7%) of the adolescents were satisfied with their lives (very satisfied and satisfied), whereas 354 (78.3%) were unsatisfied with their life. The relationship between self-rated health and life satisfaction among adolescents in Saudi Arabia was documented. The Chi-square test reveals that SRH and LS are significantly associated (χ2=10.7, p<0.001).

**Table 2.** The association between self-rated health and life satisfaction among adolescents in Saudi Arabia.

**Variables**	**Life Satisfaction**				**Total**	
**Self-Rated Health**	**Satisfied**		**Unsatisfied**			
					**N.**	**%**
	**N.**	**%**	**N.**	**%**		
**Healthy**	88	25.0	266	75.5	354	78.5
**Unhealthy**	10	10.0	88	90.0	98	21.5
**Total**	98	21.7	354	78.3	452	100

Stepwise binary logistic regression analyses were performed to determine the association between LS and SRH with physical activity among adolescents. Table 3 illustrates these analyses adjusting for adolescents’ backgrounds. Students SRH and LS were both found to be independently associated (AOR=2.5, 95%CI: 1.5-4.3, p≤0.001). Moreover, compared to non-active adolescents, adolescents who reported being moderately active and highly active were more likely to report being healthy (AOR=2.5, 95%CI: 1.5-4.8, p<0.001) and (AOR=3.4, 95%CI: 2.0-5.8, p< 0.001), respectively. Also, adolescents’ life satisfaction was independently associated with father’s education (AOR=1.8, 95%CI: 1.2–3.0, p=0.023), studying in private schools (AOR=1.9, 95%CI: 1.3–2.9, p=0.011) and being highly active (AOR=3.0, 95%CI: 1.6-4.2, p=0.007).

**Table 3.** Binary logistic regression analyses of adolescents self-rated health (SRH) and life satisfaction (LS).

**Variables**	**Model A**	**Model B**
	**SRH (Healthy)** **AOR [95%C.I.] p-value**	**LS (Satisfied)** **AOR [95%C.I.] p-value**
Age		
19–20 years (ref.)	-	-
17–18 years	1.0 [0.5 – 1.9] 0.912	1.5 [0.6 – 3.5] 0.334
15–16 years	1.6 [0.8 – 3.1] 0.137	1.8 [0.8 – 4.3] 0.146
**Fathers’ Education**		
≤12 years (ref.)	-	
>12 years	1.0 [0.7 – 1.6] 0.855	1.8 [1.2 – 3.0] 0.023
**Mothers’ Education**		
≤12 years (ref.)	-	-
>12 years	1.2 [0.7 – 1.8] 0.571	0.7 [0.4 – 1.3] 0.321
**Family style (live with)**		
Both parents (ref.)	-	-
Other	0.9 [0.4 – 1.9] 0.854	0.6 [0.3 – 1.5] 0.330
**School Type**		
Public School (ref.)	-	-
Private School	1.0 [0.6 – 1.5] 0.984	1.9 [1.3 – 2.9] 0.011
**Life Satisfaction**		
Dissatisfied (ref.)	-	NA
Satisfied	2.5 [1.5 – 4.3] 0.001	
**Self-Health Rated**		
Unhealthy (ref.)	NA	-
Healthy		2.5 [1.5 – 4.3] 0.001
**Physical Activity**		
Inactive (ref.)	-	-
Moderate active	2.5 [1.5 – 4.3] 0.001	1.1 [0.5 – 2.2] 0.764
Highly active	3.4 [2.0 – 5.8] 0.001	3.0 [1.6 – 4.2] 0.007

SRH – Self-Rated Health; LS – Life Satisfaction.

## Discussion

Health and life satisfaction can be evaluated objectively through many clinical indicators, but it can also be studied subjectively through questionnaires designed to reveal a general sense of SRH and LS [2, 7–9, 14–17]. This study aimed to investigate the relationship between general LS and SRH and its association with physical activity among Saudi adolescents while considering some potential determinants.

In this study, the logistic regression analysis, adjusting for adolescents’ socio-demographic variables, revealed that adolescents’ SRH was independently associated with their sense of LS. This finding appears to be consistent with the common expectation and information from other international studies. Results from internationally representative samples of 32 European countries indicated a positive association between LS and self-reported health status among young adolescents across all countries [21]. The relationship may move in both directions, yet it is clear that both are determinants of each other. However, adolescence is described as a period of significant physical, mental, and social changes, presenting new psychological and social development challenges; in return, these may influence their self-perception of LS, health, and well-being. The association between perceived LS and SRH among adolescents could lead to better intervention efforts to promote their health and lifelong development. Additionally, this study provided the first evidence of an association between SRH and the level of physical activity among adolescents in Saudi Arabia. The adjusted logistic regression indicated that adolescents’ SRH was independently associated with the level of physical activity. Moderately and highly active adolescents were more likely to report themselves as healthy. This study confirmed findings from previous research suggesting that SRH is associated with physical activities [16, 22–24]. Therefore, by promoting physical activities, adolescents may have opportunities to have better health and build a great sense of health status. Public health policymakers should promote the idea that physical activity can lead to a greater sense of well-being or act as a health and medical intervention. Moreover, adolescents’ LS was independently associated with adolescents’ father's high level of educaction and physical activity. High father education may reflect high socioeconomic status in adolescents’ life. Similarly, the Health Behavior in School-aged Children (HBSC) study covering 41 countries revealed that family affluence was significantly positively associated with school children’s high life satisfaction in nearly all countries [25]. In fact, highly active adolescents were three times more likely to report satisfaction with life compared to those who were inactive. Although we could not find research that addressed the association between life satisfaction and physical activity behavior in the Saudi population, prior research has investigated the association between LS and smoking behaviors among adolescents. Alsubaie, in his study, found that perceived dissatisfaction with life was associated with smoking behaviors among Saudi adolescents [19]. This finding, however, is consistent with many studies around the world. For example, life dissatisfaction is associated with several health-risk behaviors [16, 22, 24, 26].Therefore, by promoting physical activities among adolescents, they may build competencies, develop good relationships with others, and strengthen self-esteem, which might be necessary for their general health and life satisfaction. LS can be seen as an important construct in positive psychology and an individual’s quality of life. Therefore, public health policymakers, schools, communities, and parents should benefit from this finding and promote physical activity among adolescents. These findings reinforce the idea that physical activity is a healthy behavior with important consequences for health and well-being and should be considered when developing national policies to enhance a great sense of health and satisfaction with life [9]. Consequently, the present findings justify public health policies and intervention programs to increase physical activity among adolescents throughout school and school health services. Previous studies in Saudi Arabia have highlighted the importance of improving and strengthening school health and recommended that national policies and comprehensive strategies are urgently needed to promote healthy behaviors among children and adolescents [18, 19, 27–30].

The findings in the present study need to be interpreted in light of some limitations. First, because it is a cross-sectional design, causation cannot be inferred. Second, all findings were based on self-reports and therefore subjected to potential self-reporting bias. Third, in this study, LS and SRH were measured by two questions and therefore may not effectively convey the several components comprising these two issues. Fourth, findings were limited to data from male adolescent students in Riyadh city; therefore, our results may not be representative. Despite these limitations, this study offered unique findings concerning adolescents’ health and quality of life among the Saudi adolescent population. Further, the stratified random sampling and the large sample size of this presented study can partially protect against the influences of potential random error related to self-reporting.

## Conclusion

Findings from this study corroborate previous international research suggesting a positive connection between SRH and LS with physical activity. The current study showed an independent association of PA with life satisfaction and perceived health status among adolescents. Thus, an intervention designed to promote and increase physical activity could have several health benefits and increase life satisfaction among adolescents. The findings from this study provide important information for policymakers and evidence-based justification for public health policies to increase physical activity among adolescents. Future research should examine the associations between SRH and LS in more depth, taking into account other confounding variables, and more determinants of adolescent well-being should be included.

## Acknowledgements

### Conflict of interest

The authors declare that there is no conflict of interest.

### Ethics approval

The ethical approval for the study was obtained from the Ethical Review Committee of the Ministry of Education, Riyadh. The obtained official letters were delivered to the Riyadh Education Authority.

### Consent to participate

A letter explaining the purpose, method, and anticipated benefit of the study was attached to each questionnaire. Confidentiality was assured by indicating they were not requested to write their name on the questionnaire and by assuring that their responses would not in any way be linked to them.

### Personal thanks

The author would like to acknowledge the Ministry of Education, Riyadh education authority, and all school principals and teachers who facilitated the survey and extended the needed support.
